# The Influence of Cell Culture Density on the Cytotoxicity of Adipose-Derived Stem Cells Induced by L-Ascorbic Acid-2-Phosphate

**DOI:** 10.1038/s41598-019-56875-0

**Published:** 2020-01-09

**Authors:** Yuan-Kun Wu, Yuan-Kun Tu, Jiashing Yu, Nai-Chen Cheng

**Affiliations:** 1 Department of Internal Medicine, National Taiwan University Hospital and College of Medicine, Taipei, Taiwan; 2 Department of Orthopedics, E-Da Hospital/I-Shou University, Kaohsiung, Taiwan; 3 Department of Chemical Engineering, College of Engineering, National Taiwan University, Taipei, Taiwan; 4 Department of Surgery, National Taiwan University Hospital and College of Medicine, Taipei, Taiwan; 5 Research Center for Developmental Biology and Regenerative Medicine, National Taiwan University, Taipei, Taiwan

**Keywords:** Mesenchymal stem cells, Stem-cell differentiation

## Abstract

Ascorbic acid-2-phosphate (A2-P) is an oxidation-resistant derivative of ascorbic acid that has been widely employed in culturing adipose-derived stem cells (ASCs) for faster expansion and cell sheet formation. While high dose ascorbic acid is known to induce cellular apoptosis via metabolic stress and genotoxic effects, potential cytotoxic effects of A2-P at high concentrations has not been explored. In this study, the relationship between ASC seeding density and A2-P-induced cytotoxicity was investigated. Spheroid-derived ASCs with smaller cellular dimensions were generated to investigate the effect of cell-cell contact on the resistance to A2-P-induced cytotoxicity. Decreased viability of ASC, fibroblast, and spheroid-derived ASC was noted at higher A2-P concentration, and it could be reverted with high seeding density. Compared to control ASCs, spheroid-derived ASCs seeded at the same density exhibited decreased viability in the A2-P-supplemented medium. The expression of antioxidant enzymes (catalase, SOD1, and SOD2) was enhanced in ASCs at higher seeding densities. However, their enhanced expression in spheroid-derived ASCs was less evident. Furthermore, we found that co-administration of catalase or N-acetylcysteine nullified the observed cytotoxicity. Collectively, A2-P can induce ASC cytotoxicity at higher concentrations, which can be prevented by seeding ASCs at high density or co-administration of another antioxidant.

## Introduction

Ascorbic acid (AA) has been employed in various cell culture conditions for its ability to suppress the generation of reactive oxygen species^1,2^. Particularly, AA has been shown to exert beneficial effects in stem cell culture. For example, the compound enhances the generation of induced pluripotent stem (iPS) cells from both murine and human fibroblasts and facilitates the differentiation of embryonic stem cells and iPS cells into the cardiac lineage^3–5^. Moreover, AA can increase cell proliferation and DNA synthesis of mesenchymal stem cells (MSCs) during *in vitro* culture^6^.

Although supplementing AA in cell culture provides multiple benefits, a high concentration of AA increased intracellular reactive oxygen species levels via the production of hydrogen peroxide (H_2_O_2_)^7,8^. Consequently, AA at high concentrations can inhibit glyceraldehyde 3-phosphate dehydrogenase (GAPDH) and induce mitoptosis^9,10^, resulting in cellular apoptosis in cancerous cell lines. Genotoxicity was also observed at a high concentration of AA as a result of double-strand breaks due to overwhelming oxidative stress^11^. This property of AA has been leveraged in cancer cell eradication, as cancerous cells express lower levels of catalase and consequently metabolize H_2_O_2_ much slower than normal cells^12^. Since the use of AA is limited by its rapid oxidation, short half-life, and potential H_2_O_2_-induced cytotoxicity, L-ascorbic acid 2-phosphate (A2-P), a more stable derivative of AA, is widely adopted as an alternative for culturing various cell types^6,13–15^.

Adipose-derived stem cell (ASC) is an abundant source of MSCs. It exhibits excellent potential for clinical use to enhance tissue regeneration. A2-P has been shown to accelerate cell growth and prolong the lifespan of ASCs^16^. Our previous study also revealed that A2-P stimulated ASC sheet formation with enhanced ASC stemness and transdifferentiation capabilities^17^. Intriguingly, although ASCs stimulated with 250 μM A2-P exhibited higher proliferative activity relative to control ASCs, we noticed that these cells at different passages appeared to express a higher quantity of the senescence marker p21^17^. Moreover, Choi *et al*. demonstrated decreased cell proliferation when 500 μM A2-P was used for bone marrow-derived MSC culture as compared to 250 μM A2-P^6^. Therefore, we speculated that A2-P, despite more oxidation-resistance than AA, still generates low levels of H_2_O_2_ and induces sub-lethal cellular injury when added in the medium for ASC culture.

Since A2-P is a vital supplement for ASC culture to increase cell yield and fabricate cell sheets, it is crucial to determine the potential cytotoxicity associated with A2-P concentration and cell culture density. At a lower seeding density, fibroblasts exposed to increasing levels (100 to 1000 μM) of AA displayed reduced viability^18^. This phenomenon can be readily explained by the insufficient recruitment of anti-oxidative capabilities when the total cell number is low in each culture well. However, when we subjected low-density ASCs to the A2-P-supplemented medium, a drastic decrease in cell viability with marked cell death was observed. This phenomenon could not merely be attributed to the low cell number in each culture well. Therefore, we further investigated the underlying mechanism contributing to the survival of ASCs under various seeding densities and A2-P concentrations.

## Results

### A2-P induced dose-dependent cytotoxicity in ASCs

**Figure 1:**
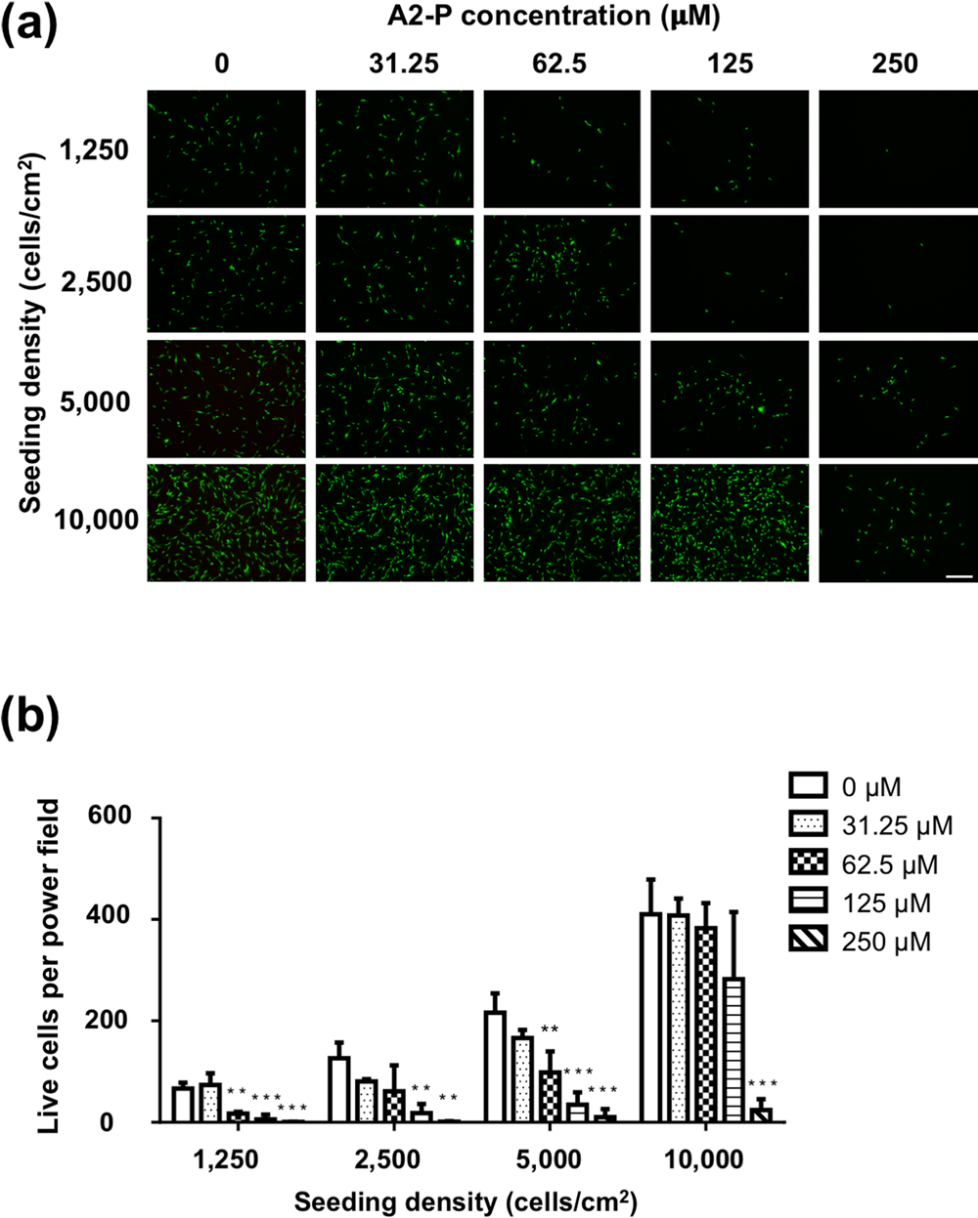
The influence of A2-P concentration and ASC seeding density on A2-P-induced cytotoxicity. (**a**) Fluorescent microscopic images showed calcein AM staining of ASCs cultured for 1 day at different seeding densities (1250, 2500, 5000, 10000 cells/cm^2^) under different A2-P concentrations (0, 32.5, 62.5, 125, 250 μM). Cells stained green were live cells. Scale bar = 100 μm. (**b**) Quantitative data of the number of live cells per high power field. At the same seeding density, increasing A2-P concentration resulted in significantly fewer stained live cells. Data are presented as mean ± SD of 3 independent experiments. *p < 0.05, ** P < 0.01, ***P < 0.001 relative to the 0 μM group of respective seeding densities.

ASCs were seeded at densities ranging from 1,250 to 10,000 cells/cm^2^, and treated with A2-P of various concentrations (0 to 250 μM) for 24 h, followed by calcein AM staining (Fig. 1a). At low seeding densities, we observed a dose-dependent relationship between A2-P concentration and viable ASC numbers per high power field. At higher cell seeding densities, a relatively high A2-P concentration was required to decrease the number of viable cells significantly. For example, at a seeding density of 1,250 cells/cm^2^, increasing the A2-P concentration to 62.5 μM significantly decreased the number of viable ASCs comparing to the control group without A2-P treatment (17.3 ± 3.5 vs. 66.7 ± 11.7 live cells/high power field, p < 0.01; Fig. 1b). When the seeding density was increased to 10,000 cells/cm^2^, only the 250 μM group exhibited significantly less viable cells relative to the control group (24.3 ± 21.5 vs. 410.0 ± 68.6 live cells/high power field, p < 0.001; Fig. 1b). This set of results was in line with the observation in Choi’s study that increasing A2-P concentration resulted in lower cell viability^6^. Moreover, increasing seeding density reverted this phenomenon and exerted a cytoprotective effect.

### A2-P induced less cell death than AA

**Figure 2:**
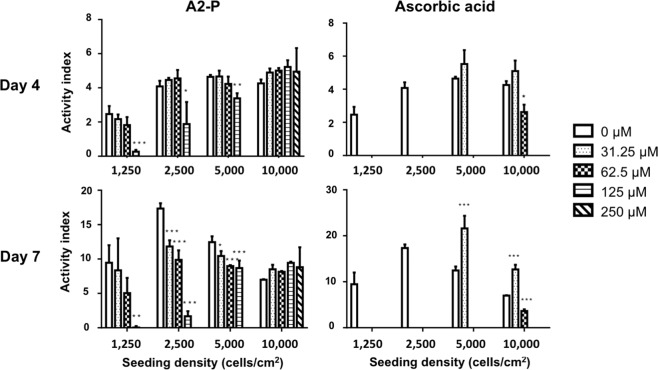
Different cytotoxic effects of A2-P and ascorbic acid exerted on ASCs. Viability of ASCs was measured at different cell seeding densities and exposed to various concentrations of A2-P or ascorbic acid for 4 and 7 days. The activity index of ASCs was defined as a ratio of fluorescent value of alamar blue relative to that of day 1. Overall, cell viability was pronouncedly lower in the ascorbic acid group relative to the A2-P group at the same concentration. Increasing concentrations of A2-P and ascorbic acid both decreased the viability of ASCs at the same seeding density. Data are presented as mean ± SD of 3 independent experiments. *p < 0.05, ** P < 0.01, ***P < 0.001 relative to the 0 μM group of respective seeding densities.

In order to estimate the cytotoxicity induced by AA or A2-P, ASC at various seeding densities was exposed to different concentrations of both compounds for 7 days. For assessment of cell proliferation, experimental results were shown as activity index, defined as the normalized ratio of viable cell number to day 1. Both A2-P and AA suppressed ASC proliferation at high concentrations. On day 4 of culturing, A2-P at higher concentrations (125 μM or 250 μM) was found to suppress the activity index of ASCs, but the effect of proliferation suppression by A2-P was nullified in the 10000/cm^2^ group even at a high A2-P concentration of 250 μM. However, AA produced a more drastic effect, quelling activity index close to zero at any concentration in the 1250 cells/cm^2^ and 2500 cells/cm^2^ groups. At low concentrations of AA (31.25 μM or 62.5 μM), ASCs at a higher seeding density of 5000 cells/cm^2^ or 10000 cells/cm^2^ exhibited the restored viability. The length of culturing days (4 or 7 days) did not alter. On day 7, the trend of the ASC activity index remained similar to that of day 4. The dose-dependent suppression of A2-P on the activity index was absent for ASCs seeded at a higher seeding density of 5000 cells/cm^2^ or 10000 cells/cm^2^ (Fig. 2).

### Fibroblasts were less susceptible to A2-P-induced cytotoxicity than ASCs

**Figure 3:**
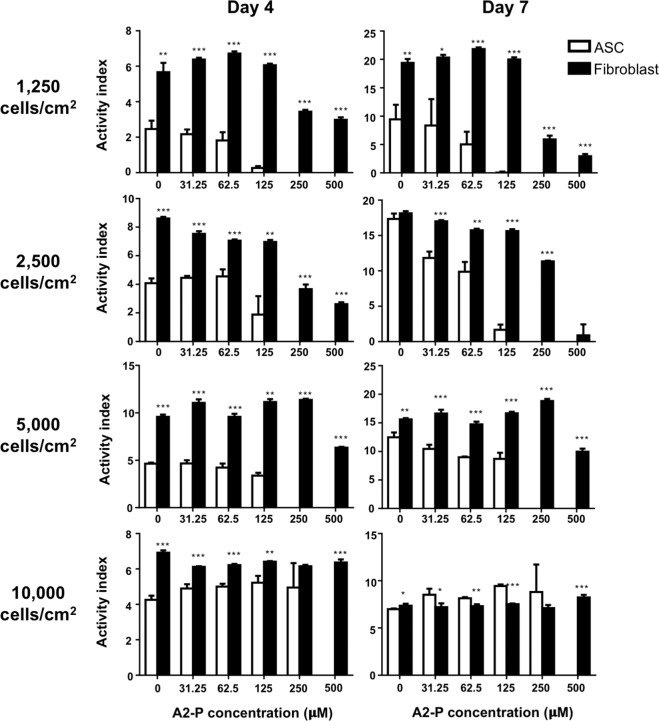
Different tolerance of ASCs and fibroblasts toward A2-P-induced cytotoxicity. Viability of ASCs or fibroblasts was measured at different cell seeding densities and exposed to various concentrations of A2-P for 4 and 7 days. The activity index was defined as a ratio of fluorescent value of alamar blue relative to that of day 1. At the same seeding density, fibroblasts exhibited relatively higher activity indexes than ASCs across all concentrations of A2-P, except at the day 7 of the 10000 cells/cm^2^ group. Data are presented as mean ± SD of 3 independent experiments. *p < 0.05, **P < 0.01, ***P < 0.001 relative to the ASC group of respective A2-P concentrations.

To elucidate the A2-P-induced cytotoxic effect among different cell types, we seeded dermal fibroblasts and ASCs at various seeding densities with various concentrations of A2-P. Similarly, fibroblasts and ASCs seeded at higher densities were able to maintain proliferative activities at higher A2-P concentrations (Fig. 3). Except for day 7 of the 10000 cells/cm^2^ group, fibroblasts generally demonstrated higher values of activity index compared to ASCs across all concentrations of A2-P. Moreover, at a high concentration of 500 μM A2-P, fibroblasts still demonstrated proliferative activity, while ASCs exhibited activity index values approaching zero, indicating a higher tolerance to A2-P-induced cytotoxicity in fibroblasts.

### Smaller ASCs were more vulnerable to A2-P-induced cytotoxicity

**Figure 4:**
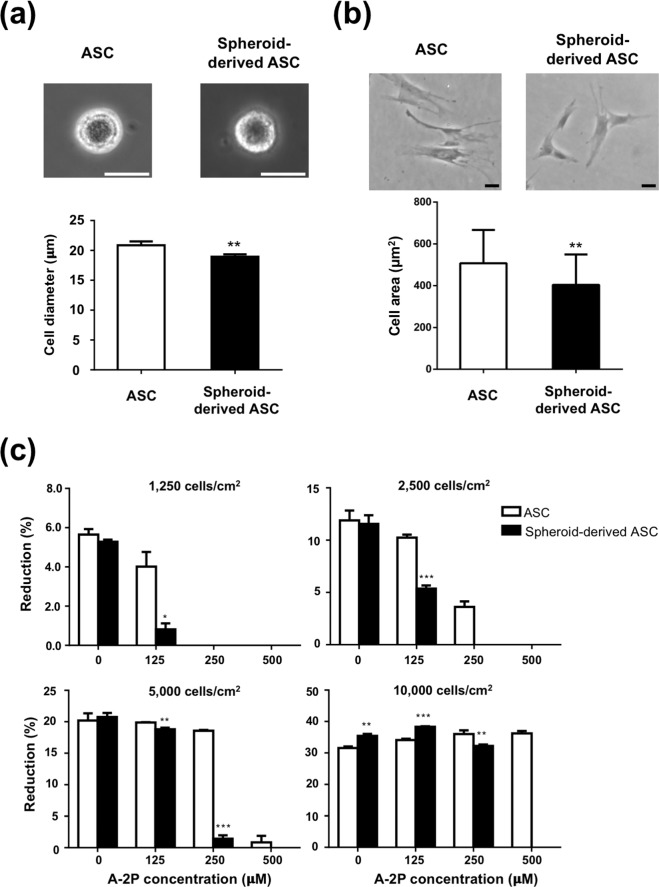
Association between ASC size and A2-P-induced cytotoxicity. (**a**) Measurement of microscopic images showed significant smaller cell size of spheroid-derived ASCs relative to ASCs. **P < 0.01; Scale bar = 20 μm. (**b**) Spheroid-derived ASCs have significantly smaller area of spread compared to ASCs. **P < 0.01; Scale bar = 20 μm. (**c**) Viability of ASCs and spheroid-derived ASCs were evaluated by alamar blue assay at different seeding densities and various A2-P concentrations for 4 days. Spheroid-derived ASCs showed significantly lower tolerance than ASCs to A2-P-induced cytotoxicity. Data are presented as mean ± SD of 3 independent experiments. *p < 0.05, **P < 0.01, ***P < 0.001 relative to the ASC group of respective A2-P concentrations.

We hypothesized that cell-cell contact might alter ASCs’ resistance to A2-P cytotoxicity. Therefore, ASCs of smaller sizes were produced, as described previously^19^, to manipulate intercellular distance indirectly. Representative microscopic images of a dissociated ASC and spheroid-derived ASC revealed their dimension difference. Quantitative measurements confirmed a significantly larger diameter of ASCs relative to spheroid-derived ASCs (20.9 ± 0.6 vs. 18.9 ± 0.4μm, p < 0.01; Fig. 4a). Subsequently, ASCs and spheroid-derived ASCs were seeded in tissue culture plates at a density of 10000 cells/cm^2^. Microscopic image analysis of ASCs and spheroid-derived ASCs were analyzed to determine the spreading area of the cells. ASCs exhibited an estimated spreading area of 507.5 ± 159.7 μm^2^, while that of spheroid-derived ASCs was 404.4 ± 145.5 μm^2^ (p < 0.01; Fig. 4b). With the cells seeded at the same density in each culture well, the intercellular distance would be affected by the spreading area of the cell. Therefore, we can assume that ASCs were closer to each other than spheroid-derived ASCs under the culture condition of the same seeding density.

After treating with A2-P at the same concentration, we observed higher cell viability (indicated by the reduction percentage of alamar blue) in ASCs relative to spheroid-derived ASCs in most combinations of seeding density and A2-P concentration (Fig. 4c).

### Cell seeding density alters the expression of antioxidant enzymes of ASCs, spheroid-derived ASCs, and fibroblasts

**Figure 5:**
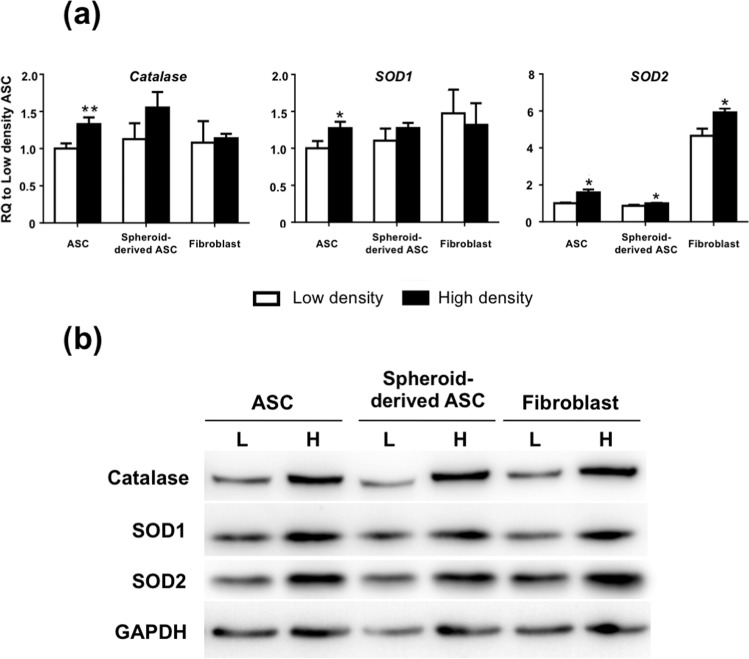
Expression of antioxidant enzymes in ASCs, spheroid-derived ASCs, and fibroblasts at low (L: 2500 cells/cm^2^) and high (H: 10000 cells/cm^2^) seeding densities. (**a**) Real-time PCR measurements for antioxidant enzymes *catalase*, *SOD1*, and *SOD2* of ASCs, spheroid-derived ASCs, and fibroblasts. ASCs exhibited significant upregulation of all three antioxidant enzymes *catalase*, *SOD1*, and *SOD2* when seeded at high density. Data are presented as mean ± SD of 3 independent experiments. *p < 0.05, **P < 0.01 relative to low-density culture condition. (**b**) Representative western blot analysis of catalase, SOD1, and SOD2 protein expression in ASCs, spheroid-derived ASCs, and fibroblasts cropped from different parts of the same gel. Full length blot is presented in Supplementary Fig. 1.

We analyzed the gene expression of antioxidant enzymes of ASCs, spheroid-derived ASCs, and fibroblasts seeded at low (2500 cells/cm^2^) and high (10000 cells/cm^2^) densities. All three types of cells displayed comparable mRNA levels of *catalase* and *SOD1*, while fibroblasts displayed higher levels of *SOD2*. ASC showed significantly higher mRNA levels of all three antioxidant enzymes at the high-density seeding condition (*catalase*: 1.33 ± 0.09-fold upregulation, p < 0.01; *SOD1*: 1.27 ± 0.09-fold upregulation, p < 0.05; *SOD2*: 1.59 ± 0.16-fold upregulation, p < 0.05, relative to the low-density seeding condition; Fig. 5a), while only mRNA level of *SOD2* was significantly increased in spheroid-derived ASC (1.16 ± 0.31-fold upregulation, p < 0.05) and fibroblasts (1.27 ± 0.35-fold upregulation, p < 0.05) relative to the low-density seeding condition. Western blot analysis was also performed, revealing that a high-density culture condition increased protein expression levels of catalase, SOD1, and SOD2, which was in line with the quantitative PCR results (Fig. 5b).

### Catalase or N-acetyl-L-cysteine rescued A2-P-induced cytotoxicity

**Figure 6:**
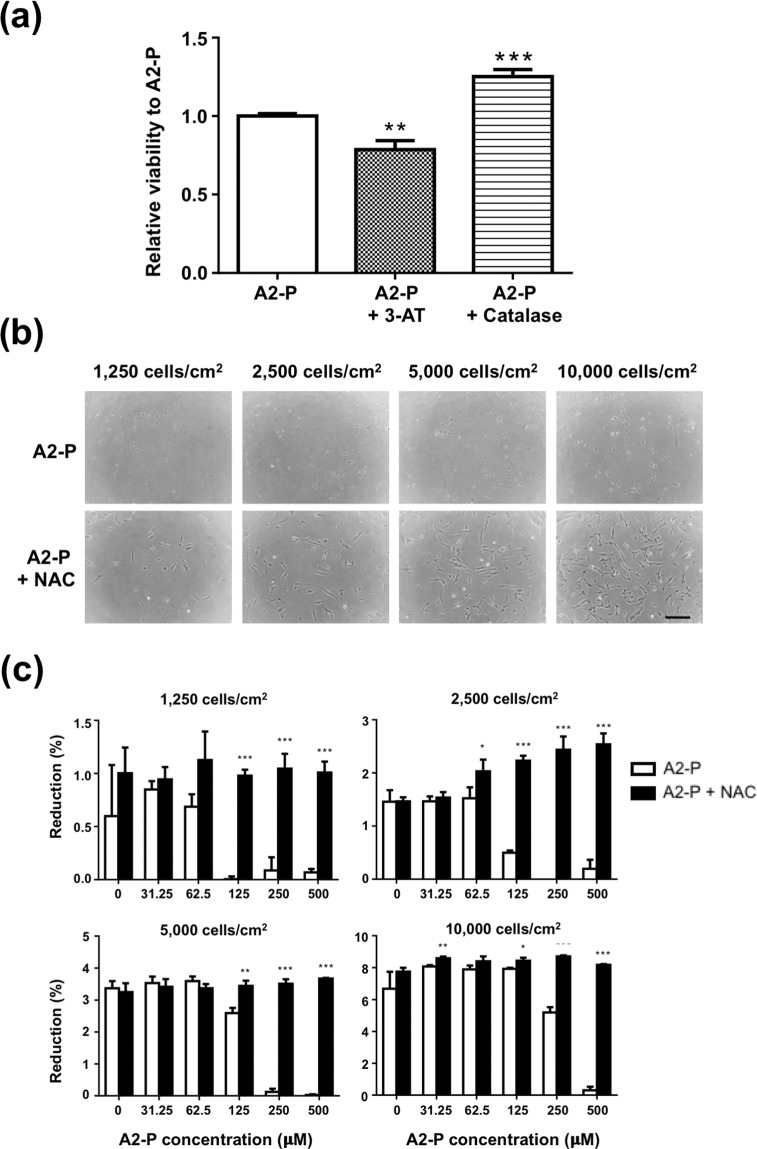
Influence of an additional antioxidant on A2-P-induced cytotoxicity. (**a**) ASCs were seeded at density of 10,000/cm^2^ and 250 μM A2-P was supplemented with or without 200 U/ml catalase for 48 h. In another group, ASCs were treated with the catalase inhibitor 20 mM 3-AT before exposing to 250 μM A2-P for 48 h. Relative viability of ASCs was estimated by alamar blue assay. Co-administration of catalase significantly increased cell viability, while pre-treatment of 3-AT decreased ASC viability compared to the A2-P-only group. **P < 0.01, ***P < 0.001. (**b**) Light microscopic images of ASCs cultured at different densities under 500 μM A2-P with or without 3 mM NAC, a ROS inhibitor. Treatment of NAC appeared to reverse the cytotoxic effect of A2-P. Scale Bar = 300 μm. (**c**) Viability of ASCs were evaluated by alamar blue assay at 1250, 2500, 5000, 10000 cells/cm^2^ with treatment of different concentrations of A2-P with or without 3 mM NAC. Co-administration of NAC reverted the decreased cell viability of A2-P across all A2-P concentrations and seeding densities. Data are presented as mean ± SD of 3 independent experiments. *p < 0.05, **P < 0.01, ***P < 0.001 relative to the A2-P group of respective concentrations.

To further investigate the relationship between catalase, an antioxidant enzyme, and A2-P, ASCs seeded at 10000/cm^2^ were pretreated with 3-amino-1,2,4-triazole (3-AT; a catalase inhibitor) or catalase before culturing in A2-P-supplemented medium. ASCs in the 3-AT- pretreated group had significantly lower relative alamar blue reduction percentage than the control group (0.79 ± 0.06-fold, p < 0.01), while the catalase-pretreated group exhibited significantly higher relative cell viability (1.25 ± 0.04-fold, p < 0.001; Fig. 6a).

N-acetyl-L-cysteine (NAC), a well-described antioxidant agent, was employed in A2-P cultures. Microscopic images of ASCs were obtained at different seeding densities cultured in 500 μM A2-P-enriched medium supplemented with or without NAC. Regardless of different cell seeding densities, more cells attached to the culture plates in the NAC-treated group after seeding, indicating higher cell viability (Fig. 6b). Moreover, when ASCs were exposed to both A2-P and NAC, NAC reversed the cytotoxic effects of A2-P across all seeding densities as indicated by the preservation of alamar blue reduction percentage. For example, in the seeding density of 10000/cm^2^ group, treatment with 500 μM NAC increased the reduction percentage of ASC from 0.3 ± 0.2% to 8.2 ± 0.1% (p < 0.001; Fig. 6c).

## Discussion

High doses of AA are known to generate H_2_O_2_ and induce cellular apoptosis. A2-P is more oxidation-resistant than AA, but potential cytotoxic effects of A2-P at high concentrations for ASC has not been well investigated. In this study, we demonstrated the dose-dependent cytotoxicity of A2-P on ASCs and showed that decreasing seeding density and increasing the intercellular distance of ASCs enhanced the cytotoxic effect of A2-P. Moreover, ASCs cultured at high density also showed enhanced expression of anti-oxidative enzymes, including catalase, SOD1, and SOD2. Therefore, in addition to the enhanced recruitment of anti-oxidative capability when the total cell number is high in high-density culture conditions, the anti-oxidative capability of each ASC is also enhanced, resulting in the nullification of A2-P-induced cytotoxicity. Since ASCs are usually cultured at a relatively high density to achieve sufficient cell number in a short time, the potential cytotoxicity induced by A2-P has been previously overlooked.

To further examine the effect of intercellular distance on the anti-oxidative capability of ASCs, we employed spheroid-derived ASCs, which were intrinsically smaller than ASCs that had been cultured continuously in conventional culture plates. The larger dimension and area of occupancy of ASCs implied shorter intercellular distances and increased cell-cell contact relative to spheroid-derived ASCs at the same seeding density. As expected, across almost all concentrations of A2-P and seeding densities tested, spheroid-derived ASCs were less tolerable to A2-P-induced cytotoxicity than ASCs. It is possible that ASCs are more resistant to A2-P than spheroid-derived ASCs because of intrinsic differences between these two types of ASCs. However, our previous study showed that these two types of ASCs were phenotypically similar in terms of cell surface marker expression and differentiation capabilities^19^. Therefore, the different responses to A2-P-induced cytotoxicity between spheroid-derived ASCs and ASCs may be attributed to reduced intercellular distances or cell-cell contact, resulting in the altered expression of anti-oxidative enzymes.

In this study, the upregulation of *catalase, SOD1*, and *SOD2* was observed in ASCs in high-density culture conditions. Moreover, inhibition of intrinsic catalase activity by 3-AT decreased ASC viability, while the addition of extrinsic catalase enhanced ASC survival in the presence of A2-P. Therefore, the importance of anti-oxidative enzymes as a protective mechanism against A2-P-induced cytotoxicity was clearly demonstrated. However, only the expression level of *SOD2* was also significantly increased in spheroid-derived ASCs and fibroblasts cultured at high density. The observation may account for the enhanced resilience of ASCs to A2-P-induced cytotoxicity relative to spheroid-derived ASCs. In addition, fibroblasts generally exhibited superior resilience to A2-P-induced cytotoxicity than ASCs, and they were found to express an appreciably higher mRNA and protein levels of SOD2. These data indicate that SOD2 may play a pivotal role in cellular protection against oxidative stress in fibroblasts. ASCs seeded at a high density of 10,000/cm^2^ exhibited increased levels of *catalase* and *SOD1* in addition to the *SOD2* anti-oxidant enzyme. The garnered antioxidation capability may explain why ASCs were later able to proliferate to the extent exceeding fibroblasts on day 7 in the conditions with A2P concentrations less than 125 μM (Fig. 3). SOD2, located primarily in the mitochondria, is vital in the maintenance of mitochondrial environment by removing superoxide anion, which is a significant factor triggering mitoptosis^20^. For example, the conditioned medium of human placental MSCs can prevent reactive oxygen species-induced cell death of endothelial cells through the upregulation of *SOD2*^21^. The protective mechanism of SOD2 primarily involves preventing the accumulation of superoxide anion close to the site of adenosine triphosphate production by facilitating the diffusion of H_2_O_2_ away from the mitochondrial matrix^22^. Downregulation of SOD2 has been implicated in numerous pathological phenotypes in metabolically active organs, such as the central nervous system and cardiovascular system^23,24^.

Seeding density, influencing intercellular contact and distance, has been shown to affect extracellular matrix production, gene expression, cytokine production, and chemotaxis of MSCs^25,26^. Reports have shown that gene and protein expression levels may be modulated via different cell-cell interactions as well as paracrine effect affected by the culture condition^27–29^. Mainly, ASCs have been studied for the influence of cell culture density on gene expression. A microarray study that systematically compared gene expression profiles of ASCs showed that proliferation-related genes were upregulated in cultures at ~50% confluence, whereas immunity and defense, cell communication, signal transduction, and cell motility genes were more highly expressed in cultures at ~90% confluence^30^. Moreover, stemness markers Nanog and c-Myc were altered in ASCs according to various seeding densities^31^. Sukho *et al*. also reported that ASC sheets of a higher cellular density increased the expression levels of vascular endothelial growth factors and FGF2, whereas specific pro-inflammatory genes such as tumor necrosis factor-α and prostaglandin synthase 2 were downregulated^32^. Our study further revealed enhanced expression of catalase, SOD1, and SOD2 were noted when ASCs were subjected to high-density culture conditions.

NAC is a well-described antioxidant agent known to reduce oxidative stress by H_2_O_2_^33^. Li *et al*. showed that co-administration of A2-P and NAC protected mitochondria from H_2_O_2_-induced oxidative stress and rescues ASCs from mitoptosis, necroptosis, and apoptosis^10^. In this study, we further showed that the addition of NAC reverted the cytotoxic effect of A2-P across all seeding densities of ASCs. The protective effect of NAC may be mediated through decreasing the BAX activation, increasing BCL2 expression, and reducing cytochrome c release from mitochondria, thus stabilizing the mitochondrial membrane^10^. Our data highlights the benefit of co-administration of A2-P and NAC for ASC culture. The information is particularly useful for ASC sheet fabrication, where a high concentration of A2-P is usually required to enhance ECM deposition^17,34^.

## Conclusions

In this study, we have demonstrated the dose-dependent effect of A2-P-induced oxidative cytotoxicity and its negative correlation with higher seeding density and smaller intercellular distance. In addition to the enhanced recruitment of anti-oxidative capabilities with more cells in high-density culture conditions, higher seeding density also exerted a defensive effect against the A2-P-induced oxidative stress by enhancing the expression of anti-oxidative enzymes. Moreover, we found that addition of another antioxidant, such as NAC, reverted the detrimental cytotoxic effect of A2-P. The observation is important for the future use of A2-P in cell cultures, particularly the ASC-associated tissue engineering applications.

## Methods

### Cell culture

Subcutaneous adipose tissue was obtained from 4 non-smoking, nondiabetic donors with an average age of 45 y (range, 32–57 y) and an average BMI of 24.6 (range, 21.0–26.6). The methods and protocol were performed in accordance with the relevant guidelines and regulations approved by the Research Ethics Committee of National Taiwan University Hospital (No. 201303038RINB), and informed consents were obtained from each participant. ASCs were isolated and characterized as described previously^35^. Briefly, the harvested adipose tissue was washed with phosphate-buffered saline (PBS; Omics Biotechnology, Taipei, Taiwan) and finely minced. The scraped adipose tissue was then placed in a digestion solution containing 1 mg/ml collagenase (Gibco, Carlsbad, CA) at 37 °C for 60 min. After digestion, the cell suspension was filtered, centrifuged and re-suspended in expansion medium. The expansion medium consisted of Dulbecco’s modified Eagle’s medium (DMEM)/F-12 (Hyclone, Logan, UT), 10% fetal bovine serum (FBS; Biological industries, Kibbutz Beit Haemek, Israel), 1% antibiotic-antimycotic (Biological Industries), and 1 ng/ml fibroblast growth factor-2 (FGF2; R&D systems, Minneapolis, MN). The cells were plated and cultured at 37 °C in a 5% CO_2_ humidified atmosphere, and the medium was changed every 2–3 days.

Fourth passage ASCs were harvested for various studies. In some experiments, human dermal fibroblasts, which were kindly provided by Dr. Tai-Horng Young from Institute of Biomedical Engineering, National Taiwan University, were used for comparison^36^. Fibroblasts were cultured in basal medium consisting of DMEM-high glucose, 10% FBS and 1% antibiotic-antimycotics till the fifth passage. When the cells have reached 90% confluence, the cells were lifted with 0.05% trypsin (Biological Industries) and re-plated. Light microscopic images were taken under Nikon Eclipse TS100 Microscope (Nikon Instruments, Tokyo, Japan) with Pentax Optio WB-2 (Pentax, Tokyo, Japan).

### Spheroid-derived ASCs

ASCs were plated onto chitosan film at a density of 1.05 × 10^5^ cells/cm^2^ using a previously described method^19^. After 3 days of culture, ASC spheroids formed on chitosan films were dissociated by HyQtase (Hyclone), transferred to new culture dishes, and expanded for 4 more days before the cells were harvested for further experiments. Cells that experienced short term spheroid formation were referred as spheroid-derived ASCs. The cell size of suspended spheroid-derived ASCs and ASCs were measured using a Scepter^TM^ 2.0 handheld automated cell counter (Merck Millipore, Burlington, MA) according to the manufacturer’s instruction. Moreover, the cell spreading area after seeding ASCs at a density of 1 × 10^4^ cells/cm^2^ were analyzed with ImageJ (v1.52k, NIH) by tracing the periphery of the cells. Light microscopic images were taken under Nikon Eclipse TS100 Microscope (Nikon Instruments, Tokyo, Japan) with Pentax Optio WB-2 (Pentax, Tokyo, Japan).

### Calcein AM staining

ASCs were seeded at different densities (1250, 2500, 5000, 10000 cells/cm^2^) in basal medium supplemented with various concentrations of A2-P (0, 32.5, 62.5, 125, 250 μM; Sigma, St. Louis, MO). After 24 h for cell attachment, ASCs were stained with the Calcein AM dye (Invitrogen) at room temperature according to the manufacturer’s protocol, and observed under a fluorescent microscope (Leica DMI6000 B). Quantification of the stained cells in the acquired microscopic images was performed by Image J (v1.52k, NIH).

### Cell viability assay

ASCs, spheroid-derived ASCs, or fibroblasts were seeded at different densities in basal medium supplemented with various concentrations of A2-P (0, 31.25, 62.5, 125, 250 μM). Ascorbic acid (AA, Sigma) of the same concentrations was also employed for ASC culture as a comparison. Alamar blue assay for cell viability assessment was performed with a protocol modified from a previous study, with the percentage of alamar blue reduction corresponding to the number of viable cells^37^. After cell seeding, an initial 24 h period was allowed for cell attachment. On days 1, 4, and 7, alamar blue (AbD Serotec, Kidlington, UK) was added into the culture medium, and the plate was further incubated at 37 °C for 2 h. The fluorescence intensity of experimental and control wells was read at 560 and 590 nm with a standard spectrophotometer (Tecan, Männedorf, Switzerland). The number of viable cells was proportional to the magnitude of dye reduction, which was expressed as the percentage of alamar blue reduction.

### Quantitative reverse transcription-PCR

Total RNAs of ASCs, spheroid-derived ASCs, and fibroblasts at low (2500 cells/cm^2^) and high (10000 cells/cm^2^) seeding densities were extracted using RNeasy Kit (QIAGEN, Hilden, Germany) according to the manufacturer’s instructions. RNA concentration was determined by optical density at 260 nm (OD_260_) using a spectrophotometer. Once RNA was isolated, complementary DNA (cDNA) was synthesized from RNA using High-Capacity cDNA Reverse Transcription Kits (Applied Biosystems, Foster City, CA). Quantitative reverse transcription-PCR was performed using iQ SYBR green supermix (Bio-Rad, Hercules, CA) and CFX Connect Real-Time PCR Detection System (Bio-Rad) for *catalase*, superoxide dismutase 1 (*SOD1*), and superoxide dismutase 2 (*SOD2*). The expression level was analyzed and normalized to GAPDH for each cDNA sample. Relative quantity (RQ) of gene expression was calculated with comparative C_T_ method. List of primers are shown in Supplementary Table 1.

### Western blot

The protein expression of catalase, SOD1, and SOD2 in ASCs, spheroid-derived ASCs, and fibroblasts at low (2500 cells/cm^2^) and high (10000 cells/cm^2^) seeding densities was determined by western blot analysis. The cells were suspended in RIPA lysis buffer (Thermo Scientific, Rockford, IL). After centrifugation, the protein content was determined in the supernatants by a bicinchoninic acid protein quantification kit (Thermo Scientific). Protein samples from ASCs or fibroblasts was added to Laemmli sample buffer and boiled for 10 min. Subsequently, proteins were separated by sodium dodecyl sulfate-polyacrylamide gel electrophoresis and blotted onto polyvinylidene difluoride membranes. Western blot was performed using anti-catalase, anti-SOD1, anti-SOD2 (all from Cell Signaling, Danvers, MA), and anti-GAPDH (Abcam, Cambridge, UK) antibodies. After overnight incubation with the primary antibodies and extensive washing, the membranes were further incubated with horseradish peroxidase-conjugated secondary antibodies for 2 h. Then the blots were developed using an enhanced chemiluminescence detection system (Millipore, Billerica, MA). Blot images were taken using the UVP BioSpectrum^®^ Imaging System (Analytika Jena, Upland, CA) with VisionWorks Analysis Software (Analytika Jena).

### Treatment with catalase, catalase inhibitor and an antioxidant

To elucidate the influence of intracellular antioxidant enzymes on the A2-P-induced cytotoxicity of ASCs, catalase (Sigma) or a catalase inhibitor 3-AT (Sigma) was used for ASC treatment. Catalase (200U/ml) was added along with 250 μM A2-P into basal medium for ASC culture for 48 h. To inhibit catalase activity, ASCs were pretreated with 20 mM 3-AT for 2 h before subjecting to A2-P treatment for 48 h. Moreover, ASCs were seeded at different densities with various concentrations of A2-P along with an antioxidant NAC (3 mM; Sigma) for 20 h. ASCs without NAC treatment were used as controls. The cell morphology was observed under a microscope, and alamar blue assay was employed to assess cell viability.

### Statistical analysis

All measurements are presented as means ± standard deviation. Statistical significance was evaluated using an independent-sample Student’s t-test or ANOVA followed by the Tukey post-hoc test. When the Tukey test was used, each group was compared to the control. All statistical analyses were performed using GraphPad Prism 7 (La Jolla, CA). Statistically significant values were defined as p < 0.05.

## Supplementary information

Supplementary Materials.

## References

[1] Taniguchi M, Arai N, Kohno K, Ushio S, Fukuda S (2012). Anti-oxidative and anti-aging activities of 2-O-alpha-glucopyranosyl-L-ascorbic acid on human dermal fibroblasts. Eur J Pharmacol.

[2] Kim JE (2008). Vitamin C inhibits p53-induced replicative senescence through suppression of ROS production and p38 MAPK activity. International journal of molecular medicine.

[3] Esteban MA (2010). Vitamin C enhances the generation of mouse and human induced pluripotent stem cells. Cell Stem Cell.

[4] Cao N (2012). Ascorbic acid enhances the cardiac differentiation of induced pluripotent stem cells through promoting the proliferation of cardiac progenitor cells. Cell research.

[5] Sato H (2006). Collagen synthesis is required for ascorbic acid-enhanced differentiation of mouse embryonic stem cells into cardiomyocytes. Biochemical and biophysical research communications.

[6] Choi KM (2008). Effect of ascorbic acid on bone marrow-derived mesenchymal stem cell proliferation and differentiation. J Biosci Bioeng.

[7] Uetaki M, Tabata S, Nakasuka F, Soga T, Tomita M (2015). Metabolomic alterations in human cancer cells by vitamin C-induced oxidative stress. Sci Rep.

[8] Clement MV, Ramalingam J, Long LH, Halliwell B (2001). The *in vitro* cytotoxicity of ascorbate depends on the culture medium used to perform the assay and involves hydrogen peroxide. Antioxidants & redox signaling.

[9] Yun J (2015). Vitamin C selectively kills KRAS and BRAF mutant colorectal cancer cells by targeting GAPDH. Science.

[10] Li CJ, Sun LY, Pang CY (2015). Synergistic protection of N-acetylcysteine and ascorbic acid 2-phosphate on human mesenchymal stem cells against mitoptosis, necroptosis and apoptosis. Sci Rep.

[11] Castro Maria, Carson Georgia, McConnell Melanie, Herst Patries (2017). High Dose Ascorbate Causes Both Genotoxic and Metabolic Stress in Glioma Cells. Antioxidants.

[12] Doskey CM (2016). Tumor cells have decreased ability to metabolize H2O2: Implications for pharmacological ascorbate in cancer therapy. Redox Biol.

[13] Shima N, Kimoto M, Yamaguchi M, Yamagami S (2011). Increased proliferation and replicative lifespan of isolated human corneal endothelial cells with L-ascorbic acid 2-phosphate. Investigative ophthalmology & visual science.

[14] Takamizawa S (2004). Effects of ascorbic acid and ascorbic acid 2-phosphate, a long-acting vitamin C derivative, on the proliferation and differentiation of human osteoblast-like cells. Cell biology international.

[15] Tsutsumi K (2012). Effects of L-ascorbic acid 2-phosphate magnesium salt on the properties of human gingival fibroblasts. Journal of periodontal research.

[16] Lin TM, Tsai JL, Lin SD, Lai CS, Chang CC (2005). Accelerated growth and prolonged lifespan of adipose tissue-derived human mesenchymal stem cells in a medium using reduced calcium and antioxidants. Stem cells and development.

[17] Yu J, Tu YK, Tang YB, Cheng NC (2014). Stemness and transdifferentiation of adipose-derived stem cells using L-ascorbic acid 2-phosphate-induced cell sheet formation. Biomaterials.

[18] Murakami K, Muto N, Fukazawa K, Yamamoto I (1992). Comparison of ascorbic acid and ascorbic acid 2-O-alpha-glucoside on the cytotoxicity and bioavailability to low density cultures of fibroblasts. Biochem Pharmacol.

[19] Cheng NC, Chen SY, Li JR, Young TH (2013). Short-term spheroid formation enhances the regenerative capacity of adipose-derived stem cells by promoting stemness, angiogenesis, and chemotaxis. Stem Cells Transl Med.

[20] Skulachev VP (2006). Bioenergetic aspects of apoptosis, necrosis and mitoptosis. Apoptosis.

[21] Liu SH (2010). Paracrine factors from human placental multipotent mesenchymal stromal cells protect endothelium from oxidative injury via STAT3 and manganese superoxide dismutase activation. Biol Reprod.

[22] Fridovich I (1998). Oxygen toxicity: a radical explanation. J Exp Biol.

[23] Srivastava V (2010). Association of SOD2, a mitochondrial antioxidant enzyme, with gray matter volume shrinkage in alcoholics. Neuropsychopharmacology.

[24] Li Y (1995). Dilated cardiomyopathy and neonatal lethality in mutant mice lacking manganese superoxide dismutase. Nat Genet.

[25] Zhou H, Weir MD, Xu HH (2011). Effect of cell seeding density on proliferation and osteodifferentiation of umbilical cord stem cells on calcium phosphate cement-fiber scaffold. Tissue Eng Part A.

[26] Przybyla L, Voldman J (2012). Probing embryonic stem cell autocrine and paracrine signaling using microfluidics. Annu Rev Anal Chem (Palo Alto Calif).

[27] Miyamoto Y, Ikeuchi M, Noguchi H, Yagi T, Hayashi S (2017). Enhanced Adipogenic Differentiation of Human Adipose-Derived Stem Cells in an *In Vitro* Microenvironment: The Preparation of Adipose-Like Microtissues Using a Three-Dimensional Culture. Cell Med.

[28] Naderi N (2014). Adipogenic differentiation of adipose-derived stem cells in 3-dimensional spheroid cultures (microtissue): implications for the reconstructive surgeon. J Plast Reconstr Aesthet Surg.

[29] Albrecht DR, Underhill GH, Wassermann TB, Sah RL, Bhatia SN (2006). Probing the role of multicellular organization in three-dimensional microenvironments. Nat Methods.

[30] Kim DS (2014). Gene expression profiles of human adipose tissue-derived mesenchymal stem cells are modified by cell culture density. PLoS One.

[31] Kim DS (2017). Cell culture density affects the stemness gene expression of adipose tissue-derived mesenchymal stem cells. Biomed Rep.

[32] Sukho P (2017). Effect of Cell Seeding Density and Inflammatory Cytokines on Adipose Tissue-Derived Stem Cells: an *in Vitro* Study. Stem Cell Rev.

[33] Aruoma OI, Halliwell B, Hoey BM, Butler J (1989). The antioxidant action of N-acetylcysteine: Its reaction with hydrogen peroxide, hydroxyl radical, superoxide, and hypochlorous acid. Free Radical Biology and Medicine.

[34] Sukho P (2018). Adipose Tissue-Derived Stem Cell Sheet Application for Tissue Healing *In Vivo*: A Systematic Review. Tissue engineering. Part B, Reviews.

[35] Cheng NC, Wang S, Young TH (2012). The influence of spheroid formation of human adipose-derived stem cells on chitosan films on stemness and differentiation capabilities. Biomaterials.

[36] Chou YS, Lin YC, Young TH, Lou PJ (2016). Effects of fibroblasts on the function of acinar cells from the same human parotid gland. Head & neck.

[37] Cheng NC, Chang HH, Tu YK, Young TH (2012). Efficient transfer of human adipose-derived stem cells by chitosan/gelatin blend films. J Biomed Mater Res B Appl Biomater.

