# Supplementary material for: The Influence of Cell Culture Density on the Cytotoxicity of Adipose-Derived Stem Cells Induced by L-Ascorbic Acid-2-Phosphate
Source: Sci Rep. 2020 Jan 9;10:104. doi: 10.1038/s41598-019-56875-0 (PMC6952413; doi:10.1038/s41598-019-56875-0)
Supplement: Supplementary file 1 — Supplementary Materials. [file 41598_2019_56875_MOESM1_ESM.docx]

**Supplemental Materials**

**The Influence of Cell Culture Density on the Cytotoxicity of Adipose-Derived Stem Cells Induced by L-Ascorbic Acid-2-Phosphate**

Yuan-Kun Wu^1†^, Yuan-Kun Tu^2†^, Jiashing Yu^3^, and Nai-Chen Cheng^4,5^*

^1^ Department of Internal Medicine, National Taiwan University Hospital and College of Medicine, Taipei, Taiwan

^2^ Department of Orthopedics, E-Da Hospital/I-Shou University, Kaohsiung, Taiwan

^3^ Department of Chemical Engineering, College of Engineering, National Taiwan University, Taipei, Taiwan

^4^ Department of Surgery, National Taiwan University Hospital and College of Medicine, Taipei, Taiwan

^5^ Research Center for Developmental Biology and Regenerative Medicine, National Taiwan University, Taipei, Taiwan

^†^ These two authors contributed equally to this work.

* Correspondence: Nai-Chen Cheng, MD, PhD

Mailing address: Department of Surgery, National Taiwan University Hospital, 7 Chung-Shan S. Rd., Taipei, Taiwan 100

E-mail address: nccheng@ntu.edu.tw

Tel: 886-2-23123456 ext. 65068; Fax: 886-2-23934358

**Supplementary Table 1.** Primer sequences used for quantitative reverse transcription-PCR analysis

| Target gene | Primer sequences | |
| --- | --- | --- |
| *Catalase* | Forward | 5’-TGGGATCTCGTTGGAAATAACAC-3’ |
|  | Reverse | 5’-TCAGGACGTAGGCTCCAGAAG-3’ |
| *SOD1* | Forward | 5’-GGTGGGCCAAAGGATGAAGAG-3’ |
|  | Reverse | 5’-CCACAAGCCAAACGACTTCC-3’ |
| *SOD2* | Forward | 5’-GACAAACCTCAGCCCTAACG-3’ |
|  | Reverse | 5’-GAAACCAAGCCAACCCCAAC-3’ |
| *GAPDH* | Forward | 5’-CAAGGCTGAGAACGGGAAGC-3’ |
|  | Reverse | 5′-AGGGGGCAGAGATGATGACC-3’ |


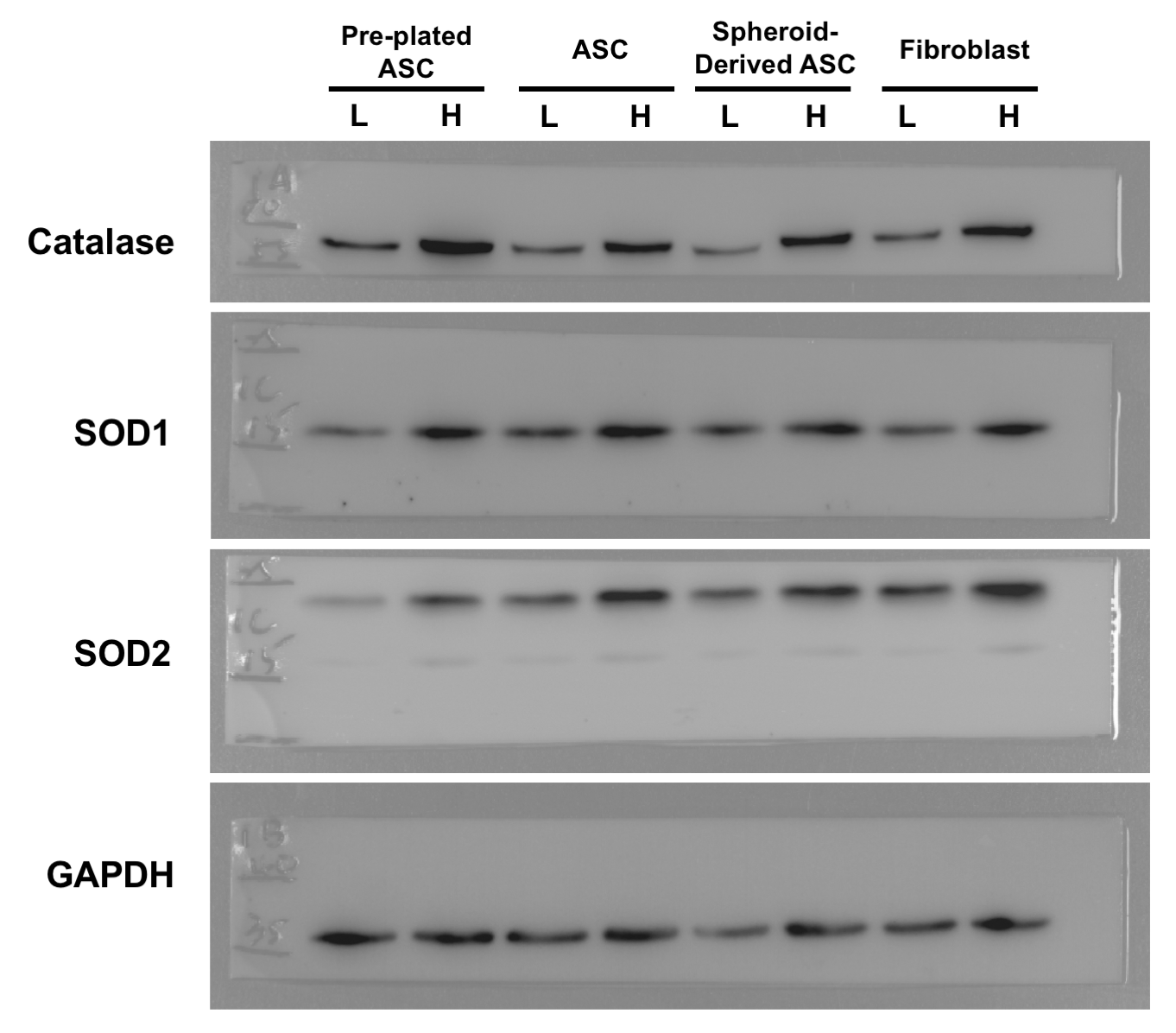


**Supplementary Figure 1.**

Original western blot gel with chemiluminescence results of various markers. “Pre-plated ASCs” were ASCs prior to plating to form ASCs or Spheroid-Derived ASCs used in the rest of the study,
